# The Challenge of Dental Education After COVID-19 Pandemic – Present and Future Innovation Study Design

**DOI:** 10.1177/00469580211018293

**Published:** 2021-06-09

**Authors:** Miguel Pais Clemente, André Moreira, João Correia Pinto, José Manuel Amarante, Joaquim Mendes

**Affiliations:** 1 Faculdade de Medicina, Universidade do Porto, Portugal; 2 Universidade do Porto, Portugal; 3 Faculdade de Engenharia, Universidade do Porto, Portugal; 4 INEGI, Porto, Portugal

**Keywords:** COVID-19, dental education, finite elements, implant dentistry, haptic systems, simulation unit, 3D printer

## Abstract

The present work suggests research and innovation on the topic of dental education after the COVID-19 pandemic, is highly justified and could lead to a step change in dental practice. The challenge for the future in dentistry education should be revised with the COVID-19 and the possibility for future pandemics, since in most countries dental students stopped attending the dental faculties as there was a general lockdown of the population. The dental teaching has an important curriculum in the clinic where patients attend general dentistry practice. However, with SARS-CoV-2 virus, people may be reluctant having a dental treatment were airborne transmission can occur in some dental procedures. In preclinical dental education, the acquisition of clinical, technical skills, and the transfer of these skills to the clinic are extremely important. Therefore, dental education has to adapt the curriculum to embrace new technology devices, instrumentations systems, haptic systems, simulation based training, 3D printer machines, to permit validation and calibration of the technical skills of dental students.

**What do we already know about this topic?**
While the engineers have been using haptic devices to control fine movements, dentist educators still not using this technology in daily practice.
**How does your research contribute to the field?**
This manuscript intends to highlighting good examples of using technology in dental education, and thus alert the decision makers to need to update the current education methods.
**What are your research’s implications toward theory, practice, or policy?**
The curriculum of Dental Education has to be changed to include the new trends that come from Engineering, as such: 3D printing, Virtual Reality, Augmented Reality, Haptic Devices, Computer Simulation.

## Introduction

During COVID period, all over the world students stopped attending the faculties as it used to be, keeping only remote videoconference classes. However, the dental teaching in a faculty has an important curriculum in clinical practice, where patients attend general dentistry. Thus, the research and innovation on dental education after COVID-19 pandemic will most probably lead to the enhancement of novel solutions and features, either based on phantoms or 3D simulators in order to maintain the dental practice.

Nevertheless, dental students can inadvertently neglect a particular aspect of safety procedures during this COVID-19 phase, where all protective measures are important. The undergraduate students are leading with these issues for the first time, like many other health care professionals and the application of rigorous procedures takes time and experience to implement.

The introduction of technology on undergraduate and post graduate dental training will provide a better efficacy on handling the lab lessons, to achieve an optimal preparation and high standards of technical hand skills. The fact that the clinical activity in dental schools could continue without patients seems somehow impossible but, with virtual simulation, this paradox might change. By joining a task force engineers and dentists will surely develop new (and improved) solutions for the dental academic community.

Perhaps at that time simulators and phantoms were created, it was just a question of innovation and technology advance, nowadays however, it is a matter of public health. The COVID-19 had a huge impact in different sectors of society, the clinics are changing and adapting to new rules to govern the dental appointments. Even in pandemic times, students in dental courses need to have a practical training. This can also be done through virtual training or 3D printed realistic models.

## The State of Art on the Introduction of Technologies for Dental Education

### Clinical Practice and Training

Before SARS-CoV-2, the curriculum of many dentistry schools in Germany was re-organized. Two key aspects of the applied changes were the integration of up-to-date teaching methods and the promotion of interdisciplinary with simulation models.^1^ The construction of models can be processed with 3D modeling software, using a 3D printing after a digital model or 3D scanning. Kroger et al. used 3 different models for distinct finalities, such as: a prosthodontic model for training veneer preparation, a conservative model for practicing dental bonding and an interdisciplinary model featuring carious teeth and an insufficient crown. The last model was evaluated in a hands-on course with 22 dental students (fourth-year), were the vast majority acknowledged the additional learning effect and support the use of simulation models for the clinical courses.^1^ However, some critics aroused, for example, the teeth, gingiva, and simulated caries all had the same color, which makes difficult the differentiation.

In the past, just 25 years ago, it was common for dental students to ask at dental clinics if they could provide extracted teeth for training. The request seemed easy accomplish, however keeping extracted human teeth is an unpleasant task due to all the preparation and disinfection procedures involved. In addition, with the advance of modern dentistry, fewer teeth are being extracted. The typodonts and the commercial houses linked to the dentistry industry started seeking for solutions were acrylic teeth appear as one alternative.

Reymus et al.^2^ using Cone Beam Computed Tomography (CBCT) digitalized extracted human teeth information and with appropriate software reproduced with a stereolithographic printer a tooth replica for endodontic training. Undergraduate students favored the availability of these replicas as they ensured training.^2^ The replicas were suitable for various aspects of endodontic training, since they show a good comparison to real teeth and exceed commercial replicas in various criteria: they are less expensive and thus produced in large number, there is a wide selection of different teeth and they have good radiopacity.^2^

One of the most important applications of 3D printing in dentistry is the implant dentistry and orofacial trauma. Frequently, they have a great contribution to plastic surgery and/or maxillofacial specialty in bone grafting procedures due to an extensive area of reconstructive surgery. Boonsiriphant et al.^3^ described an innovative technique for assisting learning in preclinical fixed prosthodontics courses with 3D printing where ideal full-contour tooth preparations are digitally scanned to the exact specifications of the ideal preparation.

Perry et al.^4^ made a review of the use of simulation in dental education, highlighting the advances in technology and communication medical simulations that are being developed to support the acquisition of requisite psychomotor skills before real-life clinical applications.^4^ For dentistry, the irreversible nature of most operative procedures means students must have the skills for safe delivery at the point of patient treatment and care. In this line, the introduction of technology such as virtual reality and haptic simulators may improve the realism of simulation and, as such, be increasingly useful in health care skill training. While supervision is required for phantom head exercises for safety reasons, associated to the use of electrically driven dental hand pieces (drills) with real burs and also for tutorial assistance and validation of procedures, the computer-generated simulations, allow dental students to repeatedly practice the same tooth preparation without the need for supervision and with real-time computer feedback.^4^ On the other hand, simulations may lack some of the realism found with phantom heads.

If in the recent past curriculum, constraints could be one of the main reasons for some kind of reluctance on the implementation of these technologies as an aid in dental education, now these solutions are more than necessaries in order to provide continuous dental training in such an area where theoretical-practical lessons are fundamental. Economical restraints may limit the investment in equipment’s as for example the Simodont (MOOG, Nieuw-Vennep, Netherlands), but more than getting new equipment, the issue is allowing the students to continue to learn in such a critical area of health sciences were the interaction with patients is fundamental.

In preclinical dental education, Phantom heads provide an efficient way to teach dental procedures safely while increasing students’ dexterity skills considerably. Modern computerized phantom head training units incorporate features of virtual reality technology and the ability to offer concurrent augmented feedback.^5^ Plessas,^5^ performed a review of 16 papers that recognized a lack of support to advise for or against the use of computerized virtual reality simulators as a replacement of the traditional phantom heads and human instruction. However, another study carried out by Mirghani et al. with a more practical view, examined the sensitivity of a haptic virtual reality dental simulator like Simodont to differences in dental training experience. Two hundred and eighty-nine participants, with 1 (n = 92), 3 (n = 79), 4 (n = 57), and 5 (n = 61) years of dental training, performed a series of tasks upon their first exposure to the simulator. They found statistically significant differences between novice (1 year) and experienced dental trainees operationalized as 3 or more years of training), but no differences between performance of experienced trainees with varying levels of experience.^6^ This research of Mirghani allowed to understand that the performance of dental students improved as their level of experience increased. Likewise, the time taken to complete the task decreased as their level of experience increased, as shown by the differences between the third, fourth, and fifth years of dental education in faculty programs.

### Automation an Instrumentation in Dentistry

The evolution has brought new words to the medical lexis such as algorithms, design, technology, robotic, force sensors, kinematics, feedback, haptic systems, 3D modeling. Wang et al.^7^ study reports the application of force feedback gloves in fields such as teleoperation and virtual reality. In order to enhance the immersive feeling of interaction with remote or virtual environments, glove-like haptic devices are used, which enable users to touch and manipulate virtual objects in a more intuitive and direct way via the dexterous manipulation and sensitive perception capabilities of human hands.^7^ In a tele-operated robotic neurosurgery scenario, haptics provides sensory stimuli that represent the interaction surgeons’ fingers.^8^ Aggravi et al.^8^ work describes the forces exerted on surgical tools by 3 neurosurgeons performing typical actions on a head phantom. The degree of realism of implemented haptic feedback related to the particular task that is being trained will have a key role in the future where the addition of haptics is believed to reduce surgical errors and potentially increase patient safety.^9^ Haptic feedback has been shown to improve the fidelity, realism and thus the training effect similar to virtual reality (VR) simulators. However, at present haptic simulators are expensive and further research as well as cost-benefit analyses of such tools must be considered to determine whether haptics is truly a surgical necessity.^10^ Even at the end of the surgical procedure, when making the stitching of a suture is possible to understand the needle insertion forces in the work of Prattichizzo et al.^11^ Experiments have shown the perception of inserting a needle using the cutaneous-only force feedback is nearly indistinguishable from the one felt by the user while using both cutaneous and kinesthetic feedback.^11^ D. Laycock and M. Day already in 2003 mentioned that a variety of haptic feedback devices were been developed and used in multiple important applications, as from joysticks used in the entertainment industry to specialized devices used in medical applications.^12^

Since restorative dentistry simulation is one of the most challenging applications involving haptics, Razavi et al.^13^ presented a paper on haptics-based tooth drilling simulator. In the specific area of maxillofacial surgery, Maliha et al.^14^ refers that training through surgical simulation is available for educational use, with haptic, physical, and web-based simulators, but there is no evidence of the benefit for maxillofacial training. Kantar et al.^15^ mentioned that cleft surgery simulators vary considerably in their features, purpose, cost, availability, and scientific evidence in support for their use. In the research carried out by Kantar et al.^15^ it was highlighted the importance of future multi-institutional collaborative initiatives that should focus on demonstrating the efficacy of current cleft simulators and developing standardized assessment scales. Even in craniofacial trauma, 3D printed haptic model appears to be an efficient low-cost support.^16^ A model has been introduced into the oral and maxillofacial surgery teaching program of undergraduate students to improve education.^16^ Reymus et al.^17^ describes the 3D printing technology, since it offers dental schools new possibilities for creating highly realistic training models covering treatment steps that have been difficult to imitate. The online platform dentaltraumaguide.org can assist recently graduated dentists in correctly handling traumatic injuries to the teeth.^17^ Nevertheless, there are still many ways in which these models could be improved, for example, modifying the quality of resins could improve the milling feeling.^18^ Commonly used model teeth are so far uniform in color and hardness, since there is no discrimination between enamel and dentin part of a tooth, therefore a printable 3D tooth with different layers for enamel and dentin was designed.^19^ Hohne et al^19^ study showed that there was a general satisfaction within the students that had the opportunity to learn a correct crown preparation on a printed tooth with different material properties for enamel and dentin. Christian Höhne and Marc Schmitter in another paper used 3D printed teeth with anatomical details for preclinical dental education.^19^ A tooth with realistic carious lesions and pulp cavity was designed, and used in 2018 with 47 dental students for preclinical education in caries excavation, direct capping of the pulp, core build-up, and crown preparation.^19^

### Translation of Research in Innovation to Dental Education

Nowadays, with the most advanced 3D-printer there is the chance to manufacture samples to emulate their geometry and material composition with high fidelity.^20^ Its capabilities, in combination with computational modeling, have provided even more opportunities for designing, optimizing, and testing the function of composite materials, in order to achieve composites of high mechanical resilience and reliability.^20^ The paper of Studart exemplifies the existing additive manufacturing technologies, which offer an attractive pathway toward the fabrication of functional materials featuring complex heterogeneous architectures inspired by biological systems.^21^ The replication in synthetic systems of design principles underlying such structural motifs has enabled the fabrication of lightweight cellular materials, strong and tough composites, soft robots and autonomously shaping structures with unprecedented properties and functionalities.^21^ With all this knowledge that science provides it is possible to obtain a 3D model, with the tooth having the resistance of dentin, enamel, or even more soft material simulating tooth decay.

Dental education will certainly reach a point where the automation processes associated to skill learning obtained by repetitive procedures and specific protocols associated to each sub-specialty in dentistry will have new instrumentation systems. These technological devices can be a reality in the future for undergraduate and post graduate education in the area of dental sciences. However, this new era of innovation has already started to be implemented in a more routinely manner for example in orthodontics. A 3D printing guide combined with digital imaging software demonstrates many advantages and is gaining more and more popularity in orthodontic treatments to achieve an accurate positioning and bonding of brackets.^22^ One of the areas in dentistry that has already a digital workflow is orthodontics, where a 3D virtual treatment planning can be used at the early stages of treatment plan.^22^ This tool has advantages for the patient, orthodontist, and the maxillofacial surgeon during the orthodontic/surgical treatment plan since it uses a single virtual anatomic model including the hard and soft tissues and teeth.^22^ To this extent, a 3D virtual surgical splints can be generated using Computer Aided Design and Manufacturing (CAD/CAM) techniques to be used during the actual surgery to help the surgeons achieve the desired results.^22^ Recent advances in computer technology and three-dimensional interactive treatment planning have made patient-specific appliance creation not only possible, but also compelling.^23^ Orthodontics and CBCT technology have evolved tremendously in the last 10 years, where the technology has gone from a predominantly diagnostic to a true clinical and translational product.^23^ The study of Nadjmi et al.^24^ presents a 3D virtual occlusion tool that calculates a realistic interaction between upper and lower dentitions to be used on preoperative planning for orthognathic surgery.

Besides orthodontics, implant dentistry is surely one of the subjects with major interest in dental education. Students should assess the first practical experiences in the field of implant dentistry at the pre-clinical stage of their education.^25^ At the Department of Prosthodontics, LMU, Munich, 120 pre-clinical students participated at the implant dentistry education course demonstrating a positive response and the general interest in this form of dental education, as well as the demand for further development in this field.^25^ Golob Deeb et al.^26^ did a pilot study with pre-doctoral students and stated that computer-aided dynamic implant navigation systems can improve implant surgical training in novice population. Research and innovation is being made in this area with new solutions in terms of different treatment on implants surfaces providing an improvement regarding the osseointegration process. The placement of self-threading endosseous dental implants can be placed in a 3D mandible and maxilla. The osteotomy utilizing standard surgical protocol with the pre-established and normal sequence of drills till the final drill corresponding to the implants diameter can be carried out. Healing abutments can also be connected to all maxillary and mandibular implants according to a one-stage implant protocol (non-submerged healing). After the implant placement surgery radiographs can be taken in order to visualize the correct placement of the implant inside the 3D mandible and maxilla corresponding to the “osseous zone.” The linear distance from the implant platform to the height of the abutment that will be used for the prosthetic rehabilitation process, can be experienced in different conditions, simulating distinct clinical situations. Likewise, the existing differences on the rehabilitation stages of implant dentistry is a constant challenge with different abutment morphology and the eventual relationship that the abutment design can have for example on marginal bone loss is of major concern for the longevity of this technique.

Understanding the loading forces on a mandibular complete denture for example, could be made with finite elements, allowing undergraduate and post graduate students for specific training with these 3D models. Revilla-León et al.^27^ had the purpose to compare the accuracy of implant analog positions on complete edentulous maxillary casts made of either dental stone or additive manufactured polymers using a coordinate measuring machine. In this case, the additive manufacturing technologies showed similar results to conventional dental stone. The dental education in such a specialized area of implant dentistry where the teaching of correct technical surgical and rehabilitation protocols is fundamental, will allow future dental implant practitioners to be able to quantify and visualize important aspects that they will surely place into practice on a daily basis when dealing with a real patient. The investment on implant education can be made taking in consideration some aspects previously mentioned or using haptic systems that can simulate the resistance of bone perforation to place implants. The more training can be offered in this area, the more demand there will be by dentists to the implant industry in order to provide new implants, new abutment solutions, offering in the future more options to the patients that seek for this kind of treatment.

At last but not the least, an area where the measurements and interactions of forces are difficult to quantify is in vivo. Finite elements method (FEM) is a valuable tool in the mechanical simulation being possible to investigate the human temporomandibular joint (TMJ) and potentially help in the future to increase the understanding of the masticatory system and the relationship between temporomandibular disorders and articular movements.^28^ Sagl et al.^28^ described a biomechanical computer simulation which is useful to investigate forces/strength/strain in complex systems such as the temporomandibular joint and disorders. A novel, dynamic computer model of the masticatory system combines a muscle driven rigid body model of the jaw region with a detailed FEM disk and elastic foundation (EF) articular cartilage, using high-resolution MRI data for protrusion and opening from the patient.^28^

The history of simulation in medical education and possible future directions was published by Bradley,^29^ where it was concluded that the quantity and quality of research in this area of medical education was limited. Such research is needed to enable educators to justify the cost and effort involved in simulation and to confirm the benefit of this way of learning in terms of the outcomes achieved through this process. Fourteen years have passed and now in 2020, it is possible to highlight the enormous effort made by many researchers, academic members of dental faculties and clinicians that want to create “added value” to dental education. However, some of the raised questions and conclusions of Paul Bradley are pertinent and up to date. The dental education has initiated to explore the innovation and new teaching concepts, however these have been made experimentally, or in an isolated manner. A dental education consensus regarding the challenge that will be placed “into our hands” due to economic, social and health issues related to the COVID-19 will accelerate the changes.

Table 1 summarizes the educational equipment and options references, according to the different target applications.

**Table 1.** List of equipment for dental health sciences education.

Conservative dentistry and prosthodontics	Simodont^®^ (Nissin Dental Products Inc., Kyoto, Japan)^30^
	DentSim (Image Navigation Ltd., New York U.S.A.)^31^
	VRDTS (Virtual reality dental training system) (Novint Technologies, Delaware, U.S.A.)
	IDSS (Iowa dental surgical simulator) (from Dentistry College of University of Iowa, U.S.A)
	Dentaroid (Nissin Dental Products, Kyoto, Japan)^32^
	Virtual dental patient (VDP)^33^
	HapTELTM (King’s College London Dental Institute and Reading University, U.K)
	VirDenT system (Faculty of Dental Medicine, University Ovidius of Constanta, Romania)^34^
	HHDTS (Handai haptic dental training system) from Japan^35^
	Falcon haptic robot^13^
	Voxelman system (from Voxel-man, Hamburg, Germany)
	Digital preparation validation tool (PVT)^36,37^
Implantology	IGI (Image guided implantology) (Image Navigation Ltd., New York, U.S.A)^38^
	Mandible/maxilla models (Navadha Enterprises/Frasaco^®^/Nissin Dental Products)
	The LMU-implant model (Frasaco^®^, Germany)^25^
	Bone Navi (Bionic Inc., Japan)
	10DR (10DR JAPAN Co., Ltd., Japan)
	Navident dynamic guidance system^26^
	Dental implant surgery simulator (DISS)^39^
Endodontics	Endo training model Castillo (VDW, Munich)
	Root canal models (Nissin Dental Products/Navadha Enterprises/Morita Corp./Real-T Endo, Acadental)
Periodontology	PerioSim©^40^
	Periodontal jaw model (Nissin Dental Products)
	Periodontal surgery model (ADEM^®^/Nacional ossos)
Maxillofacial surgery	Virtual reality – Maxillofacial simulator (VR-MFS)^41^
	Smile train cleft care simulator (from ©2019 Smile Train, Inc. Smile Train, New York, USA)
	Augmented reality toolkits^42^
	Craniofacial interactive virtual assistant Pro (CIVA Pro) (from myFace, nonprofit organization U.S.)
	Voxel dental training simulator (from Voxel-Man©)
	Touch surgery (VR system)^43^
General tools	CAD/CAM systems (Cerec/Cerec 3D/Cerec in Lab/Procera/3Shape Dental system/iTero)^44,45^
	3D-printed simulation models^16,46^
	Methacrylate blocks
	Typodonts (in phantom heads or mannequins)
	Extracted teeth
	CAVE (computer assisted virtual environment)^47^
	Virtual reality^47^
	Stereolithographic printers^17^
	CBCT
	Web based simulators^14^
	Phantom omni (Haptic system)^48^
	Machine learning prediction algorithms^49^
	Visible human project (from U.S. National Library of Medicine)

### An Insight into the Future – Dental Curriculum and Patient Treatment after COVID-19

Research is needed within the area of oral health sciences, regarding the area of this new coronavirus – SARS-CoV-2, it’s actual transmission mode, the use of personnel protective equipment and the eventual contribute that innovation and technology can offer within the dental curriculum.

On the 25th of March 2020, a survey was launched to the ADEE member institutions, involving 153 dental schools, being able to find out that in the area of non-clinical teaching, 90% of schools reported using online pedagogical software tools while the clinical based education was reported as being “very limited, with access mainly permitted for managing only emergency dental treatments or urgent dental treatments.”^50^ Bennardo et al.^51^ stated that since March 9, the School of Dentistry, Department of Health Sciences, Catanzaro, Italy, had the graduate and post-graduate students banned to attend the school and hospital, being the dental clinic only available for dental emergencies treatment. The first balance carried out in this Italian University is that clinical training cannot be totally replaced by remote activities and therefore the adjustment of these lessons will have to be recovered at the next semester.^51^

Dental medicine needs to understand the specificity of a profession involving respiratory diseases with such a high rate of transmissibility, the infectivity periods, the routes of human to human transmission and how to work safe. In order to be adequately protected and simultaneously protect the patients that attend a dental appointment, health care providers should follow the general guidelines and information provided by the world health organization, the center for disease control and prevention, their country dental association recommendations, the scientific community and the clinicians experience.

Giudice et al.^52^ highlighted the importance of testing patients for SARS-CoV-2 before attending a dental appointment. Dental care settings invariably carry the risk of 2019-nCoV infection due to the specificity of its procedures, which involves face-to-face communication with patients, and frequent exposure to saliva, blood and other body fluids, and the handling of sharp instruments.^53^ Independently to the fact that dentistry has a wide experience in infection control strategies, our perspective is that all the actions taken toward the mitigation of the COVID-19 pandemic effects are extremely important, where for example issues related to monitoring body temperature with infrared thermography can provide relevant information for the temperature assessment.

Dental clinical education depends on the patients’ attendance to a dental school or hospital university and the reduction on the number of patients is a reality due to health, economic, and social aspects. The implementation of new strategies with innovation and technology in teaching dental students with clinical activity is needed. Nowadays we are talking on the prevention and spreading of COVID-19 or new virus strains in dental settings through these contact routines, so all the efforts that can be made in the present will benefit the preparation of our students’ dental curriculum in the future.

## Conclusions

Being able to offer the best treatment, is the objective of any dental student, however dental students’ education may be compromised by the decreasing number of patients seen in clinic. Therefore, dental education will probably have to adapt the curriculum in order to maintain the quality of practical skills learned during pre and post-graduation.

By embracing new technology devices, instrumentation, haptic systems, virtual reality, simulation based training, 3D printer machines, dental students will have multiple forms of learning besides treating a patient.

Dental education should therefore understand that the time is changing, and the area of translational science between dentistry, medical doctors and engineers will surely provide future options to implement new teaching modalities. The COVID-19 forced society to change and adapt many daily processes. A pandemic that stopped the world with a general lockdown, may have contribute to the interaction of science and teaching models for a new era in dentistry.
